# Cardiovascular magnetic resonance myocardial feature tracking for the measurement of myocardial twist and untwist at rest and during dobutamine stress in healthy volunteers

**DOI:** 10.1186/1532-429X-16-S1-P14

**Published:** 2014-01-16

**Authors:** Pablo Lamata, Shazia T Hussain, Shelby Kutty, Michael Steinmetz, Jan M Sohns, Martin Fasshauer, Wieland Staab, Christina Unterberg-Buchwald, Joachim Lotz, Andreas Schuster

**Affiliations:** 1 Department of Computer Science, University of Oxford, Oxford, UK; 2 Papworth Hospital NHS Trust, Papworth Everard, Cambridgeshire, UK; 3 Children's Hospital and Medical Center, University of Nebraska College of Medicine, Omaha, Nebraska, USA; 4 Department of Pediatric Cardiology and Intensive Care Medicine and Heart Research Center, Georg-August-University, Göttingen, Germany; 5 Institute for Diagnostic and Interventional Radiology, Georg-August-University, Göttingen, Germany; 6 Department of Cardiology and Pneumology and German Centre for Cardiovascular Research, Georg-August-University, Göttingen, Germany; 7 Dept. of Biomedical Engineering, St Thomas' Hospital, King's College, London, UK

## Background

CMR feature tracking (CMR-FT) is a method of assessing strain from routinely acquired steady- state free precession (SSFP) cine images similar to echocardiographic speckle tracking. However, its application to determine myocardial twist and untwist has never previously been explored. We sought to determine the feasibility of measuring twist and untwist from routine cine images in healthy volunteers at rest and during inotropic stimulation.

## Methods

Ten healthy volunteers had routine SSFP cine images acquired at rest and after inotropic stimulation (10 and 20 micrograms of dobutamine). The rotation of the basal and apical slices, both subendocardial and subepicardial, was measured and global LV twist θ was calculated as the difference between the counter-clockwise (positive) rotation at the apex (φapex) and clockwise rotation at the base (viewed from apex), θ = φapex- φbase. Peak twist and untwist-rate and the respective times to peak were calculated using MATLAB software for both the subepicardial and subendocardial layers and compared between rest and stress.

## Results

The peak and time to peak myocardial twist and untwist-rate are displayed at the endocardial (Table 1) and epicardial levels (Table 2). Twist and untwist-rate significantly increased with faster time to peak during dobutamine stimulation at the endocardial level (p < 0.05, significance measured with a paired t-test after logarithmic transformation of the sample). These changes were paralleled by a trend towards increased twist at the epicardial level and significantly increased untwist-rate associated with faster time to peak twist and untwist-rate during dobutamine stimulation (table 2).

**Table 1:** Mean values of peak and time to peak twist and untwist-rate at the endocardial level.

Mean ± SD	Peak twist endocardial (degrees)	Time to peak twist (ms)	Peak untwist-rate endocardial (degrees/s)	Time to peak untwist-rate (ms)
Volunteers at rest	17 ± 11	393 ± 180	-140 ± 58	495 ± 194

Volunteers at 10 mcg of dobutamine	26 ± 7 (p = 0.03)	247 ± 49 (p = 0.007)	-282 ± 100 (p = 0.01)	342 ± 56 (p = 0.03)

Volunteers at 20 mcg of dobutamine	30 ± 15 (p = 0.001)	238 ± 54 (p = 0.003)	-356 ± 179 (p = 0.005)	324 ± 57 (p = 0.01)

**Table 2:** Mean values of peak and time to peak twist and untwist-rate at the epicardial level

Mean ± Standard Deviation	Peak twist epicardial (degrees)	Time to peak twist (ms)	Peak untwist-rate epicardial (degrees/s)	Time to peak untwist-rate (ms)
Volunteers at rest	12 ± 9	325 ± 106	-119 ± 57	449 ± 91

Volunteers at 10 mcg of dobutamine	17 ± 8 (p = 0.09)	257 ± 65 (p = 0.03)	-157 ± 78 (p = 0.2)	366 ± 78 (p = 0.03)

Volunteers at 20 mcg of dobutamine	18 ± 11 (p = 0.14)	204 ± 72 (p = 0.08)	-226 ± 110 (p = 0.03)	277 ± 82 (p = 0.03)

## Conclusions

It is feasible to derive myocardial twist and untwist and respective times to peak, both at rest and dobutamine stress using CMR-FT. Application of these new measures of deformation by CMR-FT should next be explored in disease states.

## Funding

German Centre for Cardiovascular Research (DZHK Partner Site Göttingen).

